# Mitochondrial Dysfunction in Bacterial Infections

**DOI:** 10.3390/pathogens12081005

**Published:** 2023-08-01

**Authors:** Nicholas M. Maurice, Ruxana T. Sadikot

**Affiliations:** 1 Department of Medicine, Division of Pulmonary, Allergy, Critical Care, and Sleep Medicine, Emory University School of Medicine, Atlanta, GA 30322, USA; 2 Atlanta Veterans Affairs Health Care System, Decatur, GA 30033, USA; 3 VA Nebraska Western Iowa Health Care System, Omaha, NE 68105, USA; 4 Division of Pulmonary, Critical Care & Sleep, Department of Internal Medicine, University of Nebraska Medical Center, Omaha, NE 68198, USA

**Keywords:** mitochondria, bacterial infection, metabolism, mitochondrial dynamics, innate immunity

## Abstract

Mitochondria are critical in numerous cellular processes, including energy generation. Bacterial pathogens target host cell mitochondria through various mechanisms to disturb the host response and improve bacterial survival. We review recent advances in the understanding of how bacteria cause mitochondrial dysfunction through perturbations in mitochondrial cell-death pathways, energy production, mitochondrial dynamics, mitochondrial quality control, DNA repair, and the mitochondrial unfolded protein response. We also briefly highlight possible therapeutic approaches aimed at restoring the host mitochondrial function as a novel strategy to enhance the host response to bacterial infection.

## 1. Introduction

Mitochondria are dynamic double-membrane-bound organelles that participate in a variety of cellular processes. In addition to serving as the site for energy production through the tricarboxylic acid (TCA) cycle, oxidative phosphorylation via the electron transport chain, and fatty acid oxidation, mitochondria are also critically involved in programmed cell death, calcium homeostasis, biosynthesis of other macromolecules, and other cellular signaling pathways. The mitochondrial network is highly dynamic and regulated, undergoing regular fission and fusion in addition to biogenesis (the creation of new mitochondria) and mitophagy (the targeted destruction of mitochondria through the process of autophagy). Further, mitochondria are unique in possessing their own mitochondrial genome of mitochondrial DNA (mtDNA) that encodes 13 proteins of the oxidative phosphorylation machinery.

Given their critical role in several cellular processes, it is not surprising that mitochondria are critically important in the host response to bacterial infection. Both intracellular and extracellular bacteria are known to target different aspects of mitochondrial biology to enhance their virulence. Cells utilize mitochondrial quality-control pathways to relieve this mitochondrial dysfunction and maintain survival. In addition, owing to their central role in regulating important signaling pathways, mitochondria also play important roles in the innate and adaptive immune response to bacterial infection. In this review, we highlight recent advances in knowledge in understanding how bacteria cause mitochondrial dysfunction by modulating several mitochondrial processes. We also briefly highlight possible therapeutic options to ameliorate pathogen-induced mitochondrial dysfunction to enhance the host defense against infection.

## 2. How Bacteria Cause Mitochondrial Dysfunction

Bacterial pathogens utilize an array of bacterial virulence factors to subvert the mitochondrial function for their own benefit. Many of these effector molecules contain mitochondrial targeting sequences and hijack the host cell’s mitochondrial import machinery to directly target the mitochondrial function [1]. Others target mitochondria through indirect mechanisms. The cellular processes targeted by these virulence factors can vary depending on the infection strategy of the bacterial pathogen. We highlight several recent discoveries in this area, focusing on how various microbial effectors modulate host mitochondrial pathways such as cellular death pathways, mitochondrial energy production, mitochondrial dynamics, mtDNA damage, quality-control pathways, and the mitochondrial unfolded protein response (UPR^mt^).

### 2.1. Cellular Death Pathways

Mitochondria play a critical role in several different cell-death pathways; most notably, the programmed cell-death pathway known as apoptosis. Upon activation of the intrinsic apoptosis pathway, characterized by the opening of the mitochondrial permeability transition pore and loss of the mitochondrial transmembrane potential (ΔΨ_m_), cytochrome c is released from mitochondria by the Bcl-2-like family proteins Bax and Bak. Cytochrome c then acts in the cytoplasm to activate caspases, which carry out the programmed cell death. In addition to their central role in the intrinsic apoptosis pathway, mitochondria also play critical roles in regulating other non-apoptotic cell-death pathways, including necroptosis and pyroptosis [2,3].

Many extracellular bacteria are known to induce apoptosis of host cells through the action of various effector proteins in host mitochondria [2]. For example, *Pseudomonas aeruginosa*, a Gram-negative bacterium responsible for a variety of infections, has several virulence factors that can contribute to host cell apoptosis [4], including pyocyanin [5], the quorum-sensing molecule N-3-oxododecanoyl homoserine lactone [6,7], and the type III secretion system (T3SS) effector protein ExoT [8]. Other bacteria similarly utilize T3SS effectors to induce mitochondrial damage and cell death [9]. For example, enteropathogenic *Escherichia coli* secretes the T3SS effectors Map and EspF, which are targeted to the mitochondria though mitochondrial targeting sequences (MTSs). Once in the mitochondria, they act to disrupt morphology, perturb calcium homeostasis, and trigger apoptosis [10,11,12]. Unlike Map and EspF, the T3SS effectors IpaD and SipD—released by *Shigella flexneri* and *Salmonella typhimurium*, respectively—do not appear to have MTSs, but still trigger cell-death pathways [9]. IpaD induces the mitochondrial depolarization and activation of the intrinsic apoptosis pathway [13]. Meanwhile, SipD appears to damage mitochondria and triggers non-canonical autophagy-mediated type II programmed cell death [14].

Numerous bacteria express pore-forming toxins (PFTs), which can broadly be classified as α-PFTs or β-PFTs, based on their pore-forming scaffold structures (α-helix or β-barrel) [15]. α-PFTs include the colicin family of proteins produced by *E. coli* and cytolysin A produced by certain bacteria from the Enterobacteriaceae family, including *E. coli* and *Salmonella enterica*. β-PFTs include *Vibrio cholerae* cytolysin (VCC), hemolysins, and leukocidins produced by *Staphylococcus aureus* as well as cholesterol-dependent cytolysins produced by several types of bacteria, including *Clostridium perfringens* (perfringolysin O), *Listeria monocytogenes* (listeriolysin), *Streptococcus pyogenes* (streptolysin), and *Streptococcus pneumoniae* (pneumolysin). These pore-forming toxins can induce calcium influx into cells, which can subsequently induce mitochondrial dysfunction and apoptosis [15,16].

Vacuolating cytotoxin A (VacA) produced by *Helicobacter pylori* is a unique PFT that can undergo limited proteolysis to yield a heterodimeric protein consisting of p34 and p58 subunits. The p34 subunit can be imported into the mitochondria inner membrane space through the translocase of the outer membrane (TOM) complex where it can assemble a β-barrel-like structure in the inner mitochondria membrane, leading to a loss of ΔΨ_m_ and apoptosis [17,18,19,20].

Bacterial porins, which are located on the outer membrane of some Gram-negative bacteria, serve important roles in regulating several processes, including the maintenance of microbial structural integrity. Interestingly, these bacterial membrane-bound protein complexes have also been implicated in the targeting of mitochondria and the induction of cell death in host cells. For example, outer membrane protein 38 (Omp38) of the bacterium *Acinetobacter baumannii* can travel to host mitochondria, leading to a release of proapoptotic cytochrome c and apoptosis-inducing factor [21]. The *Neisseria gonorrhoeae* porin PorB targets host mitochondria and utilizes TOM to translocate into the mitochondrial intermembrane space. It integrates into the inner mitochondrial membrane, where it leads to a loss of membrane potential and sensitizes cells to the induction of apoptosis by host signaling pathways [22,23,24,25]. The mechanism of how porins like PorB may travel from the outer membrane of bacteria to the mitochondria of host cells was unclear, but Deo et al. identified that *N. gonorrhoeae* utilizes outer membrane vesicles (OMVs) to target host cell mitochondria [26]. Other bacteria, including uropathogenic *E. coli* and *P. aeruginosa*, also appear to utilize OMVs to target unidentified toxins in host mitochondria to induce apoptosis [27].

Interestingly, some PFTs may induce mitochondrial dysfunction and the resultant apoptosis through mechanisms independent of their pore-forming capability. For example, non-pore-forming VCC mutants could still translocate to host mitochondria and subsequently induce mitochondrial reactive oxygen species (mtROS) generation, mitochondrial membrane permeability transitions, and the intrinsic apoptosis pathway [28]. Studies have shown that other PFTs—including Panton–Valentine leucocidin [29] and α-toxin [30] produced by *S. aureus*, pneumolysin [31], and *Clostridium difficile* toxin A [32] and toxin B [33]—can similarly act directly with mitochondrial membranes to induce mitochondrial dysfunction and cell death.

Although many extracellular bacteria have developed strategies that promote cell death among host cells, it is not surprising that many intracellular bacteria have developed survival strategies that inhibit host cell death. For example, *Anaplasma phagocytophilum*, the causative agent of human granulocytic anaplasmosis, secretes an effector, Anaplasma-translocated substrate 1 (Ats-1), which is imported into host cell mitochondria, where it blocks the induction of apoptosis [34]. Similarly, the intracellular pathogen *Chlamydia trachomatis* inhibits mitochondrial-triggered apoptosis through several proposed mechanisms, including an enhanced expression of antiapoptotic Bcl-2 proteins and a loss of proapoptotic BH3-only proteins [35,36,37,38,39,40]. Recent work has found that the *C. trachomatis* major outer membrane porin (OmpA/MOMP) may be the primary bacterial mediator responsible for the antiapoptotic effect. It is targeted to mitochondria and incorporates into the mitochondrial outer membrane where it inhibits proapoptotic BH3-only proteins [41]. The extracellular pathogen *Neisseria meningitidis* also targets an outer membrane porin, PorB, to the outer mitochondrial membrane of host cells, where it acts to inhibit apoptosis [42]. It has been postulated that the differing effects of *N. gonorrhoeae* PorB and *N. meningitidis* PorB on apoptosis may be related to their distinct membrane targets; the mitochondrial inner membrane or the mitochondrial outer membrane, respectively [2].

### 2.2. Mitochondrial Energy Production

Many bacteria are known to modulate host cell metabolic pathways as a means of enhancing bacterial survival [43]. Under most conditions, eukaryotic cells utilize glycolysis to convert glucose into pyruvate, which is then shuttled into mitochondria. Pyruvate is oxidized in the tricarboxylic acid (TCA) cycle to generate acetyl-CoA and reducing equivalents, which are used to produce ATP via the electron transport chain in the process of oxidative phosphorylation (OXPHOS). In many bacterial infections, particularly those involving intracellular pathogens, shifts in metabolic pathways have been described [43,44].

The intracellular bacterium responsible for Legionnaires’ disease, *Legionella pneumophila*, has been shown to modulate cellular metabolism. Through its type IV secretion system (T4SS), *L. pneumophila* abrogates OXPHOS and then, through unknown mechanisms, enhances cellular glycolysis, thereby promoting a metabolic shift known as the Warburg effect, which is also seen in cancer cells. This alteration favors bacterial replication, possibly by reducing antibacterial mitochondrial reactive oxygen species (mtROS) production or increasing metabolic substrates such as serine that can be utilized by intracellular bacteria [45]. This metabolic shift from OXPHOS to glycolysis has also been described with the intracellular pathogen *Brucella abortus* [46]. Similarly, the early infection of macrophages with the intracellular bacterium *Mycobacterium tuberculosis* is characterized by a hypoxia-inducible factor (HIF)-1α-mediated increase in aerobic glycolysis [47,48,49]. This initial proglycolytic phenotype, however, gives way to a late adaptation stage, characterized by oxidative metabolism and a blunted macrophage inflammatory and antimicrobial response [48]. Unlike *L. pneumophila*, *B. abortus*, and *M. tuberculosis*, *C. trachomatis* enhances OXPHOS rather than glycolysis in order to boost production of ATP that can be scavenged for rapidly dividing bacteria [50]. *Chlamydia psittaci* similarly stimulates ATP production in host cells to enhance intracellular survival [51].

*Mycobacterium leprae*, the causative agent of leprosy that infects host Schwann cells, targets several metabolic pathways to enhance survival. First, *M. leprae* increases glucose uptake in Schwann cells, but diverts glucose away from the glycolytic pathway and toward the pentose phosphate pathway to generate NADPH for lipid synthesis. In parallel, *M. leprae* attenuates the TCA cycle and OXPHOS within Schwann cell mitochondria as a mechanism to reduce oxidative stress [52,53].

Extracellular bacterial pathogens have also been demonstrated to target host metabolic pathways either directly via bacterial effectors, indirectly through bacterial processes, or as a consequence of the resultant immune response. These interactions can be complex owing to various effects from different bacterial effectors, bacterial adaptations in acute and chronic infections, and the environmental milieu at the site of infection. *P. aeruginosa* releases various effectors, including quorum-sensing molecules, phenazines, cyanide, and siderophores that attenuate mitochondrial OXPHOS [7,54]. *V. cholerae* also induces mitochondrial bioenergetic dysfunction, characterized by ROS generation through its virulence factors GbpA [55] and cholix toxin [56]. Many of the effectors that induce mitochondrial apoptosis discussed above similarly attenuate mitochondrial respiration by disrupting the mitochondrial membrane potential.

*S. aureus* is a flexible pathogen that can colonize different environmental niches in crosstalk with the host environment. *S. aureus* can induce glycolysis in host cells [57]. In chronic infection models, enhanced bacterial glycolysis can promote host cell mitochondrial stress, characterized by mtROS production and the release of the TCA cycle metabolite itaconate [27]. Host-derived itaconate is a critical metabolite that characterizes the metabolic reprogramming that occurs in bacterial infection.

Itaconate has been well recognized as an important immunometabolite generated in host macrophages that can both exert direct antibacterial effects and regulate the host response to infection. The classical activation of macrophages results in an initial upregulation in the TCA cycle and OXPHOS, followed by nitric oxide-mediated perturbations of the TCA cycle and electron transport chain. Subsequently, the upregulation of cis-aconitate decarboxylase (ACOD1) results in the generation of itaconate through the decarboxylation of the TCA cycle intermediate cis-aconitate [58]. Itaconate can directly inhibit isocitrate lyase, a bacterial enzyme required for the glyoxylate shunt pathway, thereby exerting direct antimicrobial effects on bacteria that rely on this metabolic pathway, including *Pseudomonas indigofera* [59], *M. tuberculosis* [60], *Yersinia pestis* [61], *L. pneumophila* [62], and *S. enterica* [63]. In the case of *S. enterica*, the guanosine triphosphatase Rab32 directly interacts with ACOD1 to facilitate the delivery of itaconate to *Salmonella*-containing vacuoles to kill intracellular bacteria [63]. However, numerous bacteria have evolved itaconate resistance mechanisms, including itaconate catabolism by *Y. pestis* and *P. aeruginosa* [64] and itaconate dissimilation by *M. tuberculosis* [65]. *P. aeruginosa* and *S. aureus* can also subvert itaconate toxicity in chronic infections. Both bacteria modulate microbial metabolism in response to itaconate in order to redirect carbon flux to the synthesis of extracellular polysaccharides needed for the biofilm formation utilized in persistent infections [27,66,67]. In addition to its direct antibacterial effects, itaconate has critical immunomodulatory functions that are being actively investigated and are beyond the scope of this review [58].

A virulent strain of bacterial *Klebsiella pneumoniae* (sequence type 258), a common cause of hospital-acquired pneumonia, contributes to the depletion of glucose in the airway, thereby activating host cell glutaminolysis and fatty acid oxidation. This creates an mtROS-rich microenvironment that promotes the accumulation of anti-inflammatory myeloid cells and disease tolerance necessary to establish an indolent infection [68].

*H. pylori* can also induce mitochondrial dysfunction and perturb overall cellular metabolism through the action of its virulence factor, VacA, which can induce apoptosis as discussed above. VacA inhibits the mammalian target of rapamycin complex 1 (mTORC1), a master regulator of cellular metabolism during nutrient stress, by depleting the amino acid pool within cells [19].

Although this review primarily focuses on the isolated effects of bacteria on cellular metabolism, it should be noted that there is a body of evidence that has investigated the effect of sepsis, the life-threatening organ dysfunction caused by a dysregulated host response to infection, on the bioenergetic function and metabolic pathways in host cells and tissues. Mitochondrial dysfunction has been suggested to be a major contributor to multiorgan injury in sepsis through cytopathic hypoxia. This theory, based primarily on data from animal models of sepsis, proposes that cells across various organs develop defects in OXPHOS during sepsis that prevent the utilization of oxygen for energy production [69,70]. It is unclear if this bioenergetic shutdown reflects a protective form of mitochondrial hibernation that preserves tissue oxygen levels by decreasing demand or if it is purely pathologic [71].

### 2.3. Mitochondrial Dynamics

The network of mitochondria within cells is constantly regulated by coordinated cycles of fission and fusion, collectively known as mitochondrial dynamics, which affect mitochondrial morphology and distribution. How bacterial infections induce alternations in mitochondrial dynamics is a growing area of research [72,73].

*L. monocytogenes* induces mitochondrial network fragmentation by promoting mitochondrial fission through the action of listeriolysin O [74,75] in a process that is independent of the primary mitochondrial fission protein dynamin-related protein 1 (Drp1). *L. pneumophila* similarly induces mitochondrial fission through its T4SS effector, MitF, but this process is dependent on Drp1 [45]. *H. pylori* also induces the fragmentation of host cell mitochondria through the action of Drp1 [76]. Enteropathogenic *E. coli* initially releases the T3SS effector EspZ to block mitochondrial fragmentation, but then subsequently releases EspH, which induces mitochondrial fragmentation and cell death [77]. *V. cholerae* expresses VopE, a T3SS effector, which travels to the mitochondria by hijacking the mitochondrial import machinery and interacts with the Rho GTPases Miro1 and Miro2 to block the perinuclear clustering of mitochondria normally induced by *V. cholerae* infection. Interestingly, this alteration in mitochondrial dynamics attenuates the inflammatory response in host cells [78].

*C. trachomatis* has been shown to have varying effects on mitochondrial dynamics depending on the stage of infection, promoting fusion early in infection and fission in later stages. Early infection induces mitochondrial elongation through two proposed Drp1-dependent mechanisms. First, it induces the upregulation of microRNA (miR)-30c-5p to downregulate the fission mediator Drp1 [79]. Second, it leads to enhanced cAMP-mediated phosphorylation and subsequent inactivation of Drp1 [80]. This enhanced mitochondrial fusion leads to an increase in OXPHOS and the production of ATP that can then be scavenged by the intracellular bacteria to enhance intracellular chlamydial growth [79,80]. The questions of how and why *C. trachomatis* appears to induce mitochondrial fission in later stages of infection remain unanswered [80].

The effects of *M. tuberculosis* on mitochondrial dynamics are not completely understood. One report indicates that *M. tuberculosis* induces mitochondrial fusion and enhanced ATP production in mouse-derived macrophages through the upregulation of mitofusin 1, an outer mitochondrial membrane GTPase that regulates mitochondrial fusion [81]. Another study, however, found that *M. tuberculosis* induces mitochondrial fission through degradation of the fusion regulator mitofusin 2 in human THP-1 macrophages [82].

The Gram-negative bacterium *Shigella flexneri* is normally internalized by colonic epithelial cells, where it utilizes the actin cytoskeletal network to spread to other cells. As a defense mechanism, cells employ septins, cytoskeletal proteins involved in cytokinesis, to form cage-like structures around intracellular *S. flexneri* to target the bacteria for autophagic–lysosomal destruction. The septin cage assembly process relies on a close association with the mitochondrial network. In order to escape entrapment and autophagic-mediated destruction, *S. flexneri* induces mitochondrial fission [83].

### 2.4. Mitochondrial DNA Damage

Mitochondria possess their own unique DNA, which is especially susceptible to oxidative damage from mitochondrial ROS generated during OXPHOS. Oxidatively damaged mtDNA has been increasingly recognized as an important damage-associated molecular pattern (DAMP) in bacterial infections that can be released into the cytosol, extracellular space, or bloodstream, where it can trigger inflammatory cascades when recognized by pattern recognition receptors (PRRs), including Toll-like receptors, inflammasomes, and the cyclic AMP-GMP synthase (cGAS) stimulator of interferon genes (STING) pathway. Multiple bacterial pathogens have been found to induce mtDNA damage. For example, *P. aeruginosa* has been shown to induce mtDNA damage and mtDNA release in pneumonia models, leading to acute lung injury and multiorgan failure [84,85]. *S. pneumoniae* also causes mtDNA damage and release as well as subsequent proinflammatory signaling [86,87]. Similarly, *Salmonella typhimurium* has been found to cause the cytosolic leakage of mtDNA and promote cGAS-STING signaling [88]. Both *Mycobacterium abscessus* and *M. tuberculosis* have also been found to induce mtDNA damage and mtDNA-mediated inflammatory signaling, either through the inflammasome or the cGAS-STING pathway [89,90]. *Enterococcus faecalis* also causes mtDNA instability in gastric cancer cells [91]. It remains unclear if mtDNA damage is a consistent effect of all bacterial pathogens and if augmentation of mtDNA repair pathways may be beneficial.

### 2.5. Mitochondrial Quality Control: Biogenesis and Mitophagy

The mitochondrial network is closely regulated through the quality-control processes of mitophagy; the selective autophagic destruction of damaged organelles; and biogenesis, the creation of new mitochondria. There is limited research investigating how these processes are targeted by bacteria to promote infection.

The intracellular pathogens *L. monocytogenes*, *B. abortus*, and *Mycobacterium bovis* induce mitophagy through varied mechanisms [92,93,94]. *P. aeruginosa* also induces mitophagy through the action of clustered regularly interspaced short palindromic repeats (CRISPR)-associated systems (CRISPR-Cas) [95]. *S. aureus* similarly triggered mitophagy in vitro [96] and in vivo in alveolar epithelial cells in a murine pneumonia model [97]. Conversely, *Salmonella infantis* and *Ehrlichia chaffeensis* have been shown to inhibit mitophagy in host cells [98,99].

There are various proposed mechanisms to explain why bacteria may promote or inhibit mitophagy to enhance virulence. *L. monocytogenes* enhances mitophagy to suppress mtROS that are necessary for intracellular bacterial killing, thereby enhancing survival [93]. There is a considerable overlap between the cellular machinery necessary for the selective autophagy processes of xenophagy, the selective autophagic clearance of intracellular microbes, and mitophagy. Therefore, it is not surprising that some pathogens such as *M. bovis* promote mitophagy to competitively suppress host xenophagy and increase intracellular survival [92]. Finally, *B. abortus*-mediated mitophagy plays an important role in the propagation of infection to neighboring cells through the regulation of the packaging and egress of bacteria-containing vacuoles [94].

Mitophagy plays an important role in the regulation of the inflammasome pathway through the elimination of damaged mitochondria that would otherwise be a source for mitochondrial DAMP release [100]. This fact is exploited by various bacteria to enhance their virulence. *P. aeruginosa* promotes mitophagy to decrease mtDNA-mediated inflammasome activation. This downregulation of the inflammasome may be an important contributor to the development of pseudomonal chronic infections [95,101]. Conversely, in other infections where inflammatory activation is important for pathogenicity—namely, *Salmonella infantis* enteritis and systemic infection with *Ehrlichia chaffeensis*—the downregulation of mitophagy and consequent enhanced inflammasome activation appear to be important virulence mechanisms [98,99].

Mitochondrial biogenesis was upregulated in the lungs of animal models of *S. aureus* pneumonia and this recovery process led to the resolution of lung injury [97,102]. Meanwhile, *P. aeruginosa* attenuates mitochondrial biogenesis through the downregulation of the master regulator of biogenesis peroxisome proliferator-activated receptor-γ coactivator-1α (PGC-1α) [7].

### 2.6. Mitochondrial Unfolded Protein Response

In response to mitochondrial dysfunction, cells can activate UPR^mt^ to promote the nuclear expression of genes that promote the recovery of the mitochondrial network. This is a protective transcriptional response to the accumulation of unfolded proteins that promotes adaptation and survival during mitochondrial dysfunction. Cells utilize the transcription factor ATFS-1 to monitor the mitochondrial function and adjust transcription accordingly. ATFS-1 has a nuclear localization sequence and mitochondrial localization sequence that enables it to localize to the nucleus, where it induces the transcription of genes involved in mitochondrial protein homeostasis and reactive oxygen species. While mitophagy serves to remove the most severely defective organelles from cells, UPRmt promotes the stabilization and recovery of those organelles that are salvageable [103].

By employing a *Caenorhabditis elegans* model, Pellegrino et al. investigated the relationship between UPR^mt^ and innate immunity and showed that UPR^mt^ confers long-term protection against *P. aeruginosa* by inducing the expression of a variety of innate immune genes [104]. *P. aeruginosa* initially activates UPR^mt^, but then represses the stress response through the pseudomonal acyl-CoA dehydrogenase FadE2 [88,105]. The upregulation of ATFS-1 results in the expression of antibacterial-related peptide 2 (Abf-2), *Caenorhabditis* bacteriocin (CNC-4), lysozymes, and C-type lectin 4 (clec-4) in *C. elegans* [106,107]. Whether or not this response is targeted by other bacterial pathogens is still not well understood. UPR^mt^ is not well studied in higher animals; however, its involvement in inducing immune response genes and enhancing the host response against pathogens is becoming increasingly recognized and needs to be further studied.

## 3. Mitochondrial Restoration as a Potential Therapy for Bacterial Infections

Given the seminal importance of mitochondrial dysfunction in multiple disease processes, strategies to rescue the mitochondrial function have been proposed in numerous disease contexts [73,108]. Investigations of these therapies in bacterial infections have been limited, but we highlight a few studies.

Targeting mitochondrial energy production has been investigated as a potential therapy for bacterial infections and in sepsis [109]. Hydrogen sulfide is known to reduce oxygen consumption and it has been proposed that it could induce a reversible state of mitochondrial hibernation that could be beneficial in sepsis. The infusion of the hydrogen sulfide donor NaHS reduced organ injury in a rat model of pneumococcal pneumonia. This treatment was characterized by the preservation of OXPHOS and ATP production and decreased mitochondrial damage [110]. Adenosine monophosphate-activated protein kinase (AMPK) and Sirtuin 1 act as sensors for a low cellular energy state. When activated upon mitochondrial stress, they both regulate several mitochondrial and metabolic pathways. The pharmacologic activation of both AMPK [111] and Sirtuin 1 [112] protect against organ injury in animal models of sepsis.

The activation of mitochondrial biogenesis through the heme oxygenase 1 (HO-1)–NF-E2-related factor 2 (Nrf2) pathway using inhaled carbon monoxide has been shown protect mice from *S. aureus* sepsis [113]. In a different *S. aureus* infection model, the activation of Nrf2 using rosmarinic acid led to enhanced bacterial clearance and inflammation resolution through enhanced antioxidant and mitochondrial biogenesis pathways [114]. Similarly, therapies targeting the activation of PGC-1α, a master regulator of mitochondrial biogenesis and antioxidant defenses, reduced epithelial barrier dysfunction caused by *P. aeruginosa* and decreased pathogenicity in an in vivo pneumonia model [7,115]. Targeting mitophagy has also been investigated as a potential therapy to reduce excessive inflammation in *Salmonella* infection [99].

Antioxidant therapies have long been studied as potential therapies for sepsis, with largely disappointing results in human studies. This may largely be explained by the broad distribution of these drugs and, therefore, low concentrations within mitochondria. This has spurred the development of mitochondrial-targeted antioxidants, which have been investigated as potential therapies in sepsis [109]. These molecules, such as mitoquinone (MitoQ) and mito-TEMPO, have had some promising results in animal models of sepsis [116,117,118], but need to be further investigated in infection models and human studies.

Finally, targeted therapies to enhance mitochondrial DNA repair have been investigated as potential therapies to ameliorate mitochondrial dysfunction-induced downstream effects of bacterial infection. Ogg1 is a central DNA repair enzyme involved in the repair of oxidatively damaged mtDNA. The use of a mitochondrial-targeted Ogg1 fusion protein reduced mtDNA damage, endothelial barrier dysfunction, and organ injury in an animal model of *P. aeruginosa* pneumonia [85].

## 4. Conclusions

There is a growing body of evidence that bacterial pathogens target host cell mitochondria to enhance virulence and that bacterial-induced mitochondrial dysfunction is critical for the downstream effects of infection. Bacteria can target mitochondria in overlapping mechanisms that affect mitochondrial cell-death pathways, bioenergetic pathways, mitochondrial dynamics, quality control, mtDNA damage, and the unfolded protein response. Whether therapies aimed at enhancing mitochondrial recovery following infection might provide a novel adjunctive strategy is an area of active ongoing investigation.
